# Bioelectric Potential in Next-Generation Organoids: Electrical Stimulation to Enhance 3D Structures of the Central Nervous System

**DOI:** 10.3389/fcell.2022.901652

**Published:** 2022-05-17

**Authors:** Michelle O’Hara-Wright, Sahba Mobini, Anai Gonzalez-Cordero

**Affiliations:** ^1^ Stem Cell Medicine Group, Children’s Medical Research Institute, University of Sydney, Westmead, NSW, Australia; ^2^ School of Medical Sciences, Faculty of Medicine and Health, University of Sydney, Westmead, NSW, Australia; ^3^ Instituto de Micro y Nanotecnología, IMN-CNM, CSIC (CEI UAM + CSIC), Madrid, Spain

**Keywords:** organoids model, brain, pluripotenct stem cells, electrical stimulation, CNS, bioelectricity, retina

## Abstract

Pluripotent stem cell-derived organoid models of the central nervous system represent one of the most exciting areas in *in vitro* tissue engineering. Classically, organoids of the brain, retina and spinal cord have been generated via recapitulation of *in vivo* developmental cues, including biochemical and biomechanical. However, a lesser studied cue, bioelectricity, has been shown to regulate central nervous system development and function. In particular, electrical stimulation of neural cells has generated some important phenotypes relating to development and differentiation. Emerging techniques in bioengineering and biomaterials utilise electrical stimulation using conductive polymers. However, state-of-the-art pluripotent stem cell technology has not yet merged with this exciting area of bioelectricity. Here, we discuss recent findings in the field of bioelectricity relating to the central nervous system, possible mechanisms, and how electrical stimulation may be utilised as a novel technique to engineer “next-generation” organoids.

## Introduction

The differentiation of pluripotent stem cells (PSCs) into multicellular tissue has been studied since the establishment of the first mouse embryonic stem cell lines in the 1980s (Evans and Kaufman, 1981; Martin, 1981). Stem cell technology has greatly benefited the study of the human central nervous system (CNS). Comprised of the brain, spinal cord, retina and olfactory nerve, the CNS is the most complex and critical system of the human body. Thus, as human pluripotent stem cells (hPSCs) emerged, including embryonic stem cells (ECS) and induced pluripotent stem cells (iPSCs), the CNS was a key target for differentiation.

Traditionally, the generation of different cell types *in vitro* aimed to recapitulate *in vivo* development by utilising our knowledge of developmental biology. As such, key biochemical cues, such as growth factors and signalling molecules that regulate gene expression, were employed to instruct PSCs to differentiate into various cell types. Two-dimensional (2D) monolayer cultures of neuronal and other CNS-tissues were quickly generated after the discovery of human ESCs (Thomson et al., 1998; Reubinoff et al., 2000; Reubinoff et al., 2001). However, 2D cultures lack the niche and cell-cell interactions that are crucial for CNS function. To address this, studies turned to an additional modality of differentiation: biomechanical (Eiraku et al., 2008; Eiraku et al., 2011; Eiraku et al., 2012). Biomechanical cues, such as extracellular matrix (ECM) scaffolds, alongside biochemical cues, powered the generation of three-dimensional (3D) mini-organs, or organoids, from PSCs.

Various protocols to derive organoid models of the CNS, particularly the brain and retina, have since been developed (Reviewed in Pacitti et al., 2019; Chiaradia and Lancaster, 2020; O’Hara-Wright and Gonzalez-Cordero, 2020). CNS organoids, including those derived from healthy and diseased patient-derived iPSCs, have been used to study disease and development in human tissue at an unprecedented level (Pacitti et al., 2019; Chhibber et al., 2020). As progress in our understanding of neurodevelopment is made, and the field of bioengineering evolves, differentiation protocols have been suitably optimised to generate more robust and advanced organoids. However, CNS organoids still lack many critical components and characteristics of their *in vivo* counterparts, such as long-term survival, maturing structures, and efficient oxygen transport. Moreover, differentiation protocols can be highly variable and produce heterogenous tissues (Lancaster et al., 2013). Therefore, as we look towards next-generation organoids, culture conditions must be optimised to address these shortcomings. Currently, headway is being made through fine-tuning of bioengineering approaches, such as 3D biomaterial compositions to support structural development, assembloid technology to generate wider CNS structures, or organ-on-a-chip technology to promote oxygenation (Achberger et al., 2019; Bierman-Duquette et al., 2021; Ao et al., 2022; Miura et al., 2022). However, to best replicate organogenesis *in vitro*, understanding native mechanisms in basic development is crucial.

An additional modality guiding development *in vivo*, alongside biochemical and biomechanical, is bioelectric communication (Levin, 2014; Newman, 2019). Yet, as a physiological cue, the inclusion of electrical fields in PSC-differentiation systems has been largely ignored. The role of bioelectricity *in vivo* was first elucidated in the 1800s, with studies on regeneration (Galvani, 1791; Bois-Reymond, 1884; Becker, 1961; Becker and Spadaro, 1972; Borgens et al., 1977; 1979a). Now, emerging evidence implicates bioelectricity in other key biological processes, including development, and differentiation. Low voltage endogenous electric fields (EFs) can be identified in the embryonic CNS during development and the importance of bioelectricity in guiding neural development has been demonstrated in animal models, primary neural cultures, and stem-cell derived cultures of the CNS (Metcalf and Borgens, 1994; Metcalf et al., 1994; Shi and Borgens, 1995). Control of ion flux, through manipulation of ion channels, or application of exogenous electrical stimulation, can be used to manipulate EFs both *in vivo* and *in vitro.* Meanwhile, in the field of regenerative medicine, exogenous application of low voltage electrical stimulation has been used to promote CNS regeneration, or prevent degeneration, in the clinic and experimental studies (Fu et al., 2015; Sehic et al., 2016; Marquez-Chin and Popovic, 2020; Hellenbrand et al., 2021; Krauss et al., 2021; Sanie-Jahromi et al., 2021; Sui et al., 2021; Karamian et al., 2022). Bioengineering has interfaced with bioelectricity to deliver exogenous electrical stimulation through conductive biomaterial polymers in basic 3D models of the CNS (Bierman-Duquette et al., 2021). These studies suggest promise in the use of EFs to improve organoid research and differentiation approaches. This could have major implications in the utility of organoids for applications requiring precisely controlled differentiation or high-throughput, such as drug screening. Yet, the use of advanced conductive polymers extends beyond the realm of basic cell biology and detracts from the intrinsic self-organisation capacity of PSC-derived organoids. Many of the biological processes EFs are implicated to control *in vivo*, such as proliferation and survival, are similar to those that need to be controlled *in vitro* to improve CNS organoid differentiation. Therefore, enormous potential lies with the utilisation of exogenous electrical stimulation as a powerful, non-chemical and non-genetic tool to improve organoid cultures.

In this Review, we will firstly discuss the current state of PSC-derived CNS organoids, focusing particularly on the brain, and the approaches taken to guide differentiation of CNS tissue *in vitro*. We will then discuss the role of bioelectricity and endogenous EFs *in vivo*, and the lessons we have learnt about the role of bioelectricity in the CNS. Next, we will briefly review the importance of bioelectricity and endogenous EFs in key biological processes, before highlighting the key findings from studies which have manipulated EFs *in vivo* and *in vitro*. We will discuss the pertinence of these bioelectric-driven phenomena to improving 3D organoid cultures and describe the key few studies implementing bioelectricity and bioengineering in 3D CNS tissues. Finally, we will outline future studies that must be conducted to best utilise bioelectricity for organoid engineering. These include the proper evaluation of stimulation in various differentiation protocols and omics characterisation to understand mechanisms.

## Recapitulating Central Nervous System Development in the Dish

**FIGURE 1:**
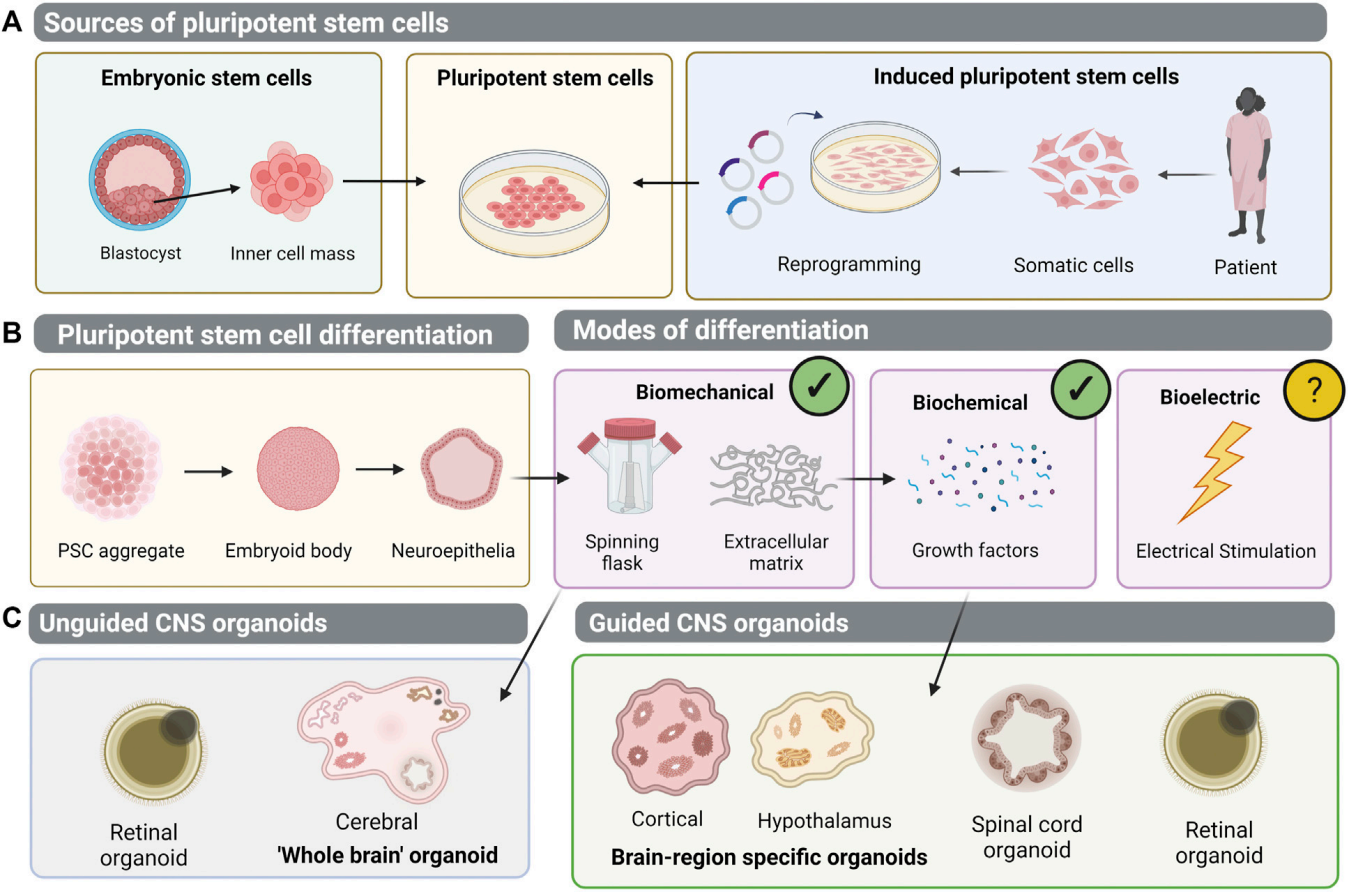
Generation of pluripotent stem cell-derived organoid models of the central nervous system (CNS). **(A)** Human pluripotent stem cells (hPSCs) can be isolated from the inner cell mass of an embryo- embryonic stem cells (ESCs). Alternatively, patient-dervied somatic cells can be reprogrammed to induced pluripotent stem cells (iPSCs). **(B)** Pluripotent stem cells can be differentiated into 3D aggregates which form embryoid bodies and neuroepithelial vesicles. Biomechanical and biochemical differentiation cues have been implemented to generate 3D organoid structures, but bioelectric cues of differentiation remain unexplored. **(C)** In unguided CNS organoid protocols, differentiation relies on the intrinsic self-organisation properties of PSCs, to generate cerebral brain organoids or retinal organoids. Meanwhile, guided protocols employ exogenous growth factors to generate brain-region specific organoids, spinal cord organoids, or retinal organoids.

The vertebrate CNS encompasses the brain, spinal cord, neural retina and olfactory nerve (Northcutt, 1984). Distinct from the peripheral nervous system, the CNS acts as a processing centre for most necessary bodily functions. Conditions affecting the CNS present an increasing global disease burden. In recent years, neurological disorders have been the foremost cause of disability-adjusted life years, and the second leading cause of death (Feigin et al., 2019). The study of the human CNS has been greatly limited by the inability to study tissue anatomy *in vivo* and tissue scarcity *ex vivo*. Whilst model organisms provide invaluable insights into CNS biological processes and mechanisms of disease, the complexity of neural networks and retinal architecture in humans remains unrivalled in comparison to lower order animals (Gibson, 1938; Uga and Smelser, 1973; Zhao and Bhattacharyya, 2018; Desmoulin-Canselier and Moutaud, 2019). The advent of PSC technology has allowed generation of human CNS derivatives in the dish (Figure 1A).

Soon after the establishment of the first human ESC line in 1998, differentiation of neuronal cultures was achieved (Thomson et al., 1998; Reubinoff et al., 2000; Reubinoff et al., 2001). Despite the complexity of neurogenesis, PSC-derived monolayer cultures were shown to readily and faithfully differentiate into a neuroectoderm lineage with accordingly dynamic and stage-specific gene expression profiles (Yeo et al., 2013; Wu et al., 2010; Fathi et al., 2011). Moreover, the default differentiation path of PSCs was identified to be neural (Eiraku et al., 2008). Transcriptomic analysis confirmed hESC-derived neuronal cultures resembled *in vivo* human neurons, particularly of the foetal brain (Pai et al., 2012). Thus, even in conditions of minimal growth factors, the biochemical cues required to drive neuronal differentiation are somewhat intrinsically provided by endogenous signalling of PSCs. However, to produce alternative CNS-derivative cells from PSCs, suitable biochemical cues were required. For example, culturing in the presence of epidermal growth factor (EGF) and fibroblast growth factor (FGF)-2, known to promote proliferation of glial precursor cells, and timed addition of tri-iodothyronine (T3), which induces oligodendrocytes, was used to derive glial cell lineages (Murray and Dubois-Dalcq, 1997; Brüstle et al., 1999; Reubinoff et al., 2001).

Similarly, by mimicking the biochemical controls governing *in vivo* embryonic head development, antagonism of Wnt and BMP with DKK1 and Noggin promoted an anterior neural fate of PSCs (Glinka et al., 1997; Mukhopadhyay et al., 2001; Banin et al., 2006; Lamba et al., 2006). Later addition of IGF-1, known to induce ectopic eye formation in animal models, differentiated cells further to a retinal lineage (Pera et al., 2001; Richard-Parpaillon et al., 2002; Lamba et al., 2006). However, 2D monolayer cultures could never replicate the complexity of the *in vivo* CNS. The cytoarchitecture and interactions of networks of cells within their 3D environment is critical to the function of the brain, spinal cord, retina and olfactory structures, both as individual structures and an integrative system. Therefore, in attempting to better model CNS tissue in the dish, the development of 3D hPSC-derived organoids was pivotal. The pioneering work of Yoshiki Sasai demonstrated that PSCs grown in suspension, rather than 2D monolayers, form embryoid body aggregates which acquire a neural fate tissue-autonomously (Eiraku et al., 2008). The group cultured mouse PSCs in suspension without serum, forming embryoid bodies, before allowing them to re-aggregate in a 96-well plate to form 3D tissue of neuroectodermal origin. This neuroectodermal-like epithelium differentiated into multi-layered cortical tissue, containing progenitor cells and neurons (Eiraku et al., 2008). Serum-free culture of embryoid body-like aggregates with quick-reaggregation, or SFEBq, thereby formed the basis of early 3D neural differentiation protocols, and the predecessor to the CNS organoid. Notably, it was the consideration of the physical microenvironment, by transitioning the cultures to suspension, that mediated the formation of this 3D tissue. Soon afterwards, modification of the SFEBq method with the addition of Matrigel to provide ECM, lead to the generation of neuroepithelial cysts which self-organised into optic cups resembling the *in vivo* retina (Eiraku et al., 2011). These may be considered the first true CNS organoid: 3D retinal structures with stratified neuroepithelia containing all major retinal cell types. The authors considered the complex forces controlling embryonic retina development and the sequential invagination and evagination folding processes (Eiraku et al., 2011; Eiraku et al., 2012). Matrigel, primarily containing laminin, aided the creation of a basement membrane structure for the aggregate to invaginate around. The inclusion of a biomechanical stimuli thereby facilitated organotypic-like development *in vitro* (Figure 1B) (Eiraku et al., 2011; Eiraku et al., 2012). Sasai’s group subsequently demonstrated differentiation of human PSC cultures to retinal and various neural 3D structures. With temporal addition of growth factors, agonists or inhibitors of FGF, Wnt, and BMP signalling networks, differentiations of forebrain, neocortex and hippocampus lineages were achieved (Kadoshima et al., 2013; Muguruma et al., 2015; Sakaguchi et al., 2015). Studies have therefore demonstrated competence of PSC-derived organoid cultures to respond to both biochemical and biomechanical stimuli, and through diversification of these cues, organotypic cultures of different CNS structures are derived, including the brain, spinal cord and retina (Figure 1B).

### Guided Approaches to Brain Organoid Differentiation

Brain organoid protocols can be broadly classified into two approaches- guided and unguided (Figure 1C). In guided approaches, building on the work of Sasai, exogenous patterning molecules are added to guide differentiation to the desired neural lineage (Eiraku et al., 2008; Eiraku et al., 2011). This has permitted brain-region specific organoid generation, including forebrain, midbrain, and hypothalamus (Qian et al., 2016; Xiang et al., 2017; Qian et al., 2018; Sloan et al., 2018; Cederquist et al., 2019; Kim et al., 2019). For example, manipulation of Sonic Hedgehog signalling patterns forebrain organoids into dorsal and ventral subdomains, better mimicking *in vivo* topology (Cederquist et al., 2019). Treatment with brain-derived neurotrophic factor (BDNF), glial cell line–derived neurotrophic factor (GDNF) and ascorbic acid guides differentiation to the midbrain and brainstem lineage (Eura et al., 2020). Sequential BMP and SMAD inhibition and activation promotes continued differentiation and maintenance of dopaminergic neurons within midbrain organoids (Jovanovic et al., 2018). Alternatively, at the neuroectodermal stage, addition of WNT3A and Hedgehog pathway agonist, Purmorphamine, induces the hypothalamic lineage (Qian et al., 2016).

### Unguided Approaches to Brain Organoid Differentiation

Unguided approaches, however, depend on the spontaneous self-organisation capacity of PSC-derived aggregates. Lancaster and Knoblich generated the cerebral, or “whole-brain” organoid, self-patterned PSC-derived aggregates embedded in Matrigel (Figure 1C) (Lancaster and Knoblich, 2014). These are heterogenous structures, comprised of multiple neural cell types within discrete regions. The addition of Matrigel for ECM provides a scaffold for neuroepithelial budding, and these buds may expand and migrate and assume polarity to differentiate into various brain regions, including forebrain, midbrain, hindbrain, choroid plexus, and retina. Single cell RNAseq confirmed the identity of neural progenitor cells, astrocytes, oligodendrocyte-like precursors and excitatory neurons, along with photosensitive retinal cells (Quadrato et al., 2017). After 4 weeks *in vitro*, cerebral organoids show regional identities, such as FOXG1 demarcating the forebrain, whilst expression of TTR delineates the choroid plexus and FZD9 the hippocampal-like region (Lancaster and Knoblich, 2014; Quadrato et al., 2017).

### Thinking Outside of the Brain: Retina, Spinal Cord, Olfactory

In vertebrates, the retina and olfactory nerve are considered an extension of the CNS. The retina is a multi-layered tissue comprising of seven cell subtypes, each highly specialised to their respective role in the light-induced phototransduction cascade. After the initial demonstration of the first CNS organoid, using the SFEBq method, retinal organoid protocols have evolved and diversified (Eiraku et al., 2011). Similarly to the brain, organoid differentiation approaches can be guided, with temporal addition of small molecules and factors informed from studies of *in vivo* retinal development (Meyer et al., 2011; Kuwahara et al., 2015; Mellough et al., 2015; Singh et al., 2015). Other groups rely more on self-patterning of PSCs, employing minimal additional factors at early stages (Reichman et al., 2014; Zhong et al., 2014; Gonzalez-Cordero et al., 2017; Reichman et al., 2017).

Resulting organoids mimic *in vivo* developmental timeline and lamellar organisation and have informed knowledge of human retinal development (Reviewed in O’Hara-Wright and Gonzalez-Cordero, 2020). Retinal organoids are also amiable to longer-term maintenance than brain organoids, and electrophysiology and calcium-mediated functionality has been described (Zhong et al., 2014; Mellough et al., 2015; Reichman et al., 2017; Gagliardi et al., 2018; Cowan et al., 2020; Saha et al., 2022). Technology towards modelling the olfactory system *in vitro*, on the other hand, remains underdeveloped. Recently, three-dimensional culture of primary olfactory progenitor cells has been achieved with Matrigel encapsulation and guided differentiation (Ren et al., 2021). However, the generation of true olfactory organoids has not yet been demonstrated.

The other major component of the CNS, the spinal cord, is responsible for neurotransmission between the interconnecting motor cortex and medulla oblongata of the brain (Bican et al., 2013). The spinal cord co-ordinates highly organised neural circuits, and morphogenesis is accordingly complex. Study of the spinal cord *in vivo* in humans is greatly hampered by inaccessibility, making it an ideal target for *in vitro* recapitulation. Spinal cord organoids have been derived *via* adaptation of guided brain organoid protocols, implementing Matrigel encapsulation and spinning flasks (Hor et al., 2018). Patterning of spinal cord organoids mimics *in vivo* axes and morphogen gradients, which is not observed within 2D cultures (Hor et al., 2018; Winanto et al., 2019). Optimised guided protocols implementing small molecules, particularly fine-tuning of BMP4 to mimic *in vivo* development, also guides spinal cord organoid morphogenesis *in vitro* (Duval et al., 2019). Resultant spinal cord organoids generate a range of cell sub-types.

Albeit the remarkable process in the field of CNS organoids and some demonstrations of functionality, the generation of a multi-organ broader CNS system in the dish has not been possible and maturation is still limited. Therefore, we are still a very long way from truly recapitulating CNS development *in vitro*. Engineering the next-generation of organoids may require turning to an additional modality to direct differentiation. It is widely established that bioelectricity, along with biochemical and biomechanical signals, plays an important developmental role (Levin, 2012; Levin, 2014; Levin, 2021). Yet studies of PSC-derived CNS organoids have not considered the role of bioelectric cues in CNS morphogenesis and attempted to recapitulate these in the dish. Recently, there has been a renewed interest in the phenomena of bioelectric-driven processes, particularly relating to nervous system development and neural differentiation (Bertucci et al., 2019; Lancaster, 2019; Bierman-Duquette et al., 2021). It may now be pertinent to consider the body of research relating to intrinsic electric fields in development, and previous studies applying electric fields to stem-cell derived cultures. By reviewing the lessons learnt from these studies, alongside the specific shortfalls in current organoid models, bioelectricity could be exploited to engineer next-generation CNS organoids.

## Biological Electrical Fields *In Vivo*: the Transepithelial Battery

**FIGURE 2:**
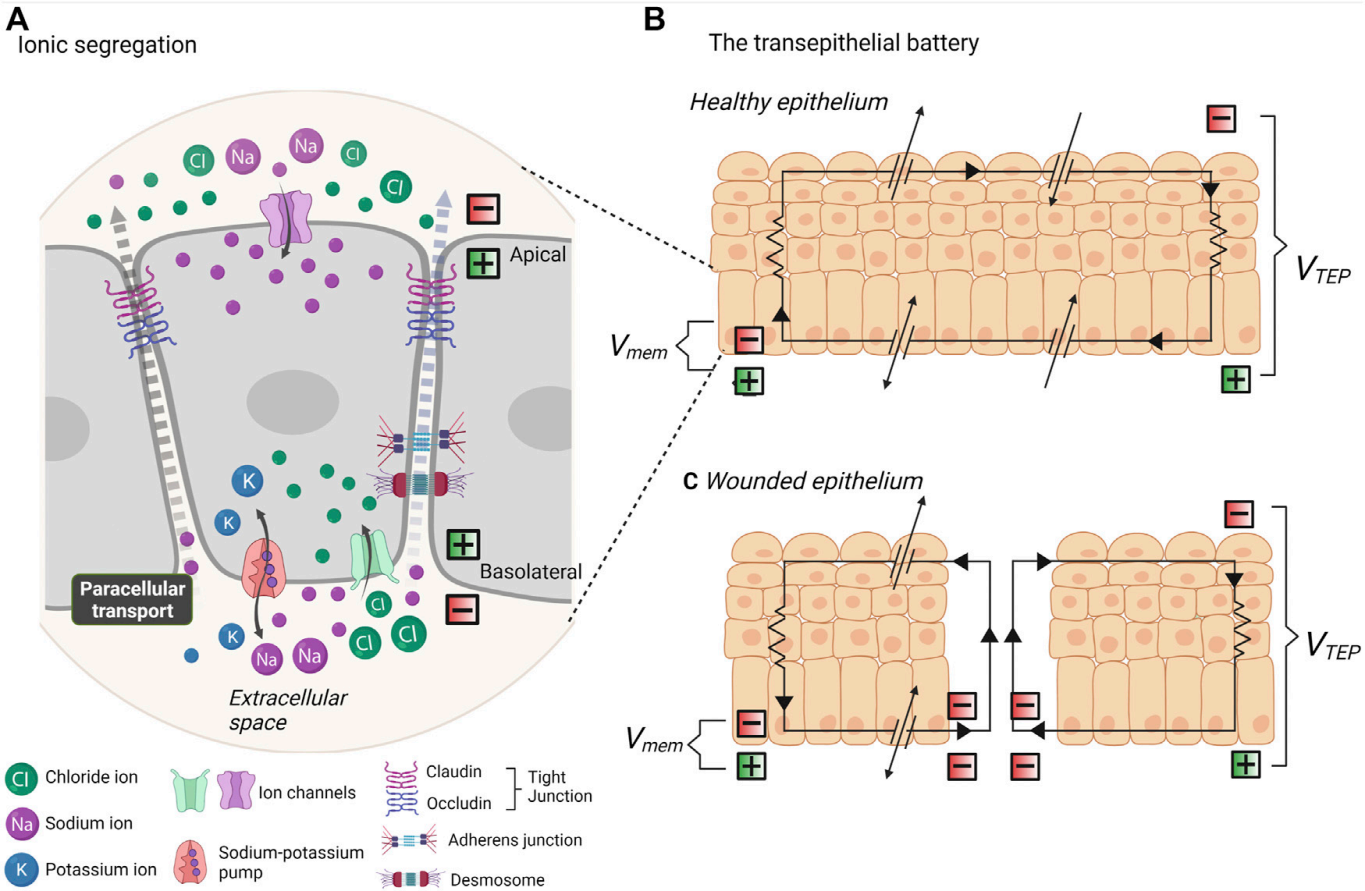
Endogenous electrical fields and the transepithelial battery. **(A)** *In vivo*, all cells exhibit a resting membrane potential due to the segregation of ionic charges. Ion transport channels are asymmetrically distributed across apical and basal membranes. Ion flux occurs transcellularly and ions are selectively transported across the plasma membrane, whilst paracellular transport (between cells) must work against the resistance of tight junctions, adherens junctions and desmosomes. **(B)** The segregation of ionic charges creates a difference in membrane potential (Vmem) at each apical-basal membrane. All of the segregated chargers in epithelia amount to a transepithelial potential difference (VTEP). **(C)** When the epithelium is breached, the VTEP drops and ionic current flows out from the site of wound. Thus, the epithelial works as a battery to mediate wound repair.

*In vivo*, cells exhibit endogenous electrical fields (EFs). Ions, and other charged biomolecules are segregated between the extra- and intracellular spaces by impermeable membranes. Ions can flow within the cell (trans-cellularly) in an apical-basal direction and be selectively transported through ion channels embedded in the membrane (Figure 2A). The asymmetric distribution of ion channels at the apical and basal membranes creates a specific pattern of ions and biomolecules and distinct domains of charges. This creates a difference in the electrical potential between the cell membrane and extracellular space, termed a membrane potential (Vmem) (Figure 2B).

In contrast to rapidly firing action potentials in excitable cells, endogenous EFs are steady or slow changing gradients present in all cell types. As low magnitude potentials, these are typically close to the resting neuron potential of ∼ −70 mV in differentiated cells, and lower yet in embryonic cells (McCaig et al., 2009). This Vmem of every cell in an epithelium amounts to a transepithelial potential difference (TEPD). Ionic current flows across the cell, transcellularly, to the extracellular space, and returns between the cells, paracellularly. The presence of tight junctions at the apical side of the intercellular space provides a barrier to ion flux. Thus, we can think of an epithelium like a battery (Figure 2B). A TEPD results from ionic segregation which is sustained by ionic pumps and channels, and these work against the resistance of tight junctions, and lesser so adherens junctions. The paracellular junctions therefore typically maintain a positive current basolaterally, and the level of resistance encountered at the junctions determine the TEPD, within a typical range of 15–60 mV (McCaig et al., 2009; Montalbetti and Fischbarg, 2009; Dubé et al., 2010). This intrinsic bioelectricity was early identified for involvement in development, regeneration and wound healing. Functional investigation established the existence of small endogenous EFs at the regeneration bud, or blastema- the mass of undifferentiated cells at amputated amphibian limbs (Becker, 1961). Limb regeneration was determined to be dependent on electrochemical activity, and enhancement or introduction of EFs promotes regeneration, even in typically non-regenerative amphibian and mammalian tissue (Becker, 1961; Becker and Spadaro, 1972; Borgens et al., 1977; Borgens et al., 1979a; Borgens et al., 1979b). Remarkably similar and steady endogenous EFs to those present in the amphibian blastema were also identified in the blastema of the amputated digit of human children (Illingworth and Barker, 1980). Known as the injury potential, or demarcation current, a difference in electrical potential is created between intact epithelium and the site of a wound (Jia et al., 2021). The TEPD at the site of injury falls and current “leaks” out from the wound edge, being the pathway of least resistance, creating an electrical field (Figure 2C). Whilst regeneration can be defined as the recreation of tissues in response to damage or degeneration, development refers to the morphogenetic transformation of the embryo to differentiated tissue. However, biologically speaking, development and regeneration require the same processes: the division, migration, and differentiation of pluripotent cells into multicellular tissues and organs. It therefore follows that EFs have a pivotal role in embryogenesis. In vertebrates, gradients of electric field activity are identifiable in the very early two-cell stage embryo (Levin et al., 2002). The first description of endogenous electrical currents in embryology was given in day 2–4 chicken embryos (Hotary and Robinson, 1990). Using a vibrational probe, an EF of 20 mV/mm was measured at the site of the presumptive hindgut (Hotary and Robinson, 1990). This stage of development co-occurs with the degeneration of gut intestinal epithelium, thus, as in the case of a wounded epithelium, providing a pathway of less resistance for leakage of current. It was soon thereafter established that endogenous EFs play a crucial role in the morphogenesis of the CNS. In the axolotl embryo, an endogenous EF of 10 mV/mm is generated from under the neural plate, polarising the embryo along the rostral/caudal axis during neurulation (Metcalf et al., 1994; Shi and Borgens, 1995). As the architecture of the neural tube takes forms, the neural folds provide a pathway of lesser resistance for current flow. Remarkably, in the *Xenopus,* artificial manipulation of transmembrane potential is sufficient to induce ectopic eye formation during early embryogenesis (Pai et al., 2012; Blackiston and Levin, 2013). Modification of endogenous EFs during neurulation by as little as 5–25 mV/mm, through application of artificial voltage, results in developmental abnormalities (Metcalf and Borgens, 1994). Of note, this effect is stage-dependent, and exposure to artificial EFs at the earlier gastrula stage does not impact embryo development. This suggests that timed EF exposure, similarly to spatiotemporal distribution of biochemical and biomechanical stimuli, has a powerful ability to guide morphogenesis.

## Harnessing Electrical Fields *In Vivo* and *In Vitro*

**FIGURE 3:**
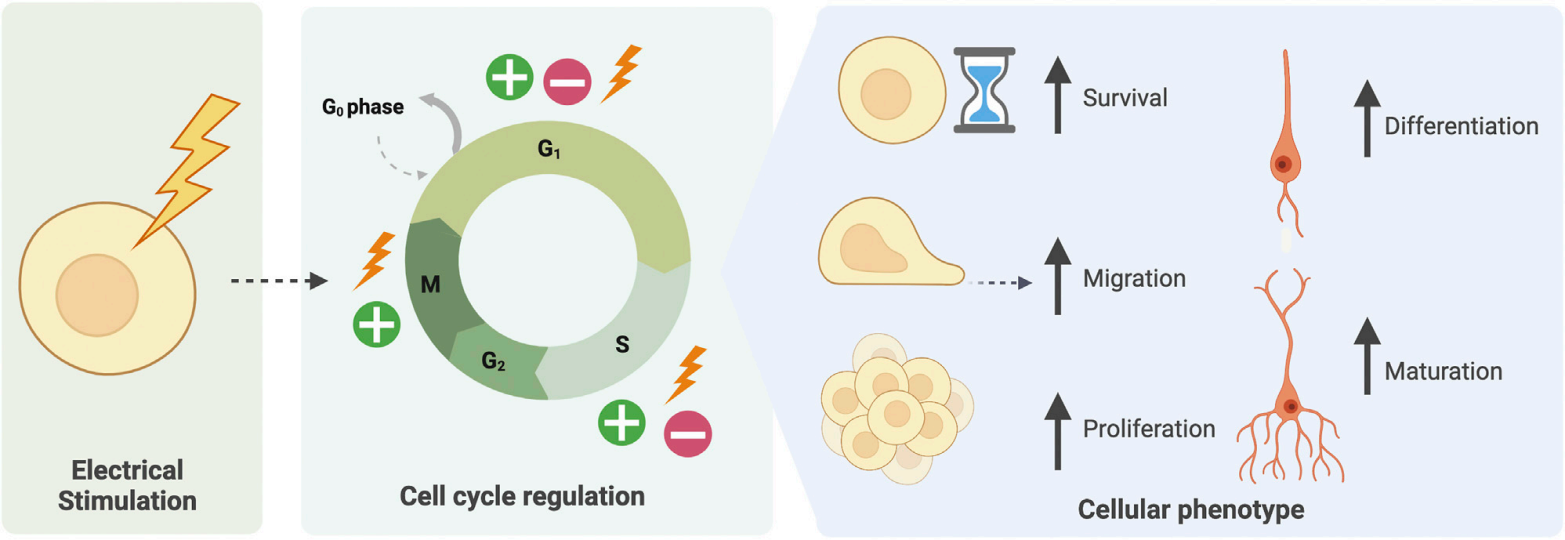
Electrical stimulation of cells mediates cell-cycle related phenotypes. Electrical stimulation of CNS cells both *in vivo* and *in vitro* has been found to regulate the cell cycle at various phases. This may lead to an increase in cell survival, migration, proliferation, or alternatively exiting the cell cycle to increase cell differentiation and maturation.

Accordingly, differentiation of PSCs could benefit from the consideration of EFs. Whilst endogenous EFs exist *in vivo,* it is also possible to apply exogenous EFs, *via* electrical stimulation, or alter endogenous EFs *via* manipulation of ion channels. The parameters of electrical stimulation, namely waveform, intensity, frequency and duration can be varied according to purpose. Numerous studies demonstrate EF modulation to target the CNS both *in vivo* in animal models, clinical studies, or *in vitro* in cell cultures (Sehic et al., 2016; Bertucci et al., 2019; Lancaster, 2019; Mobini et al., 2019; Krauss et al., 2021; Sanie-Jahromi et al., 2021). By increasing or decreasing EFs, studies have demonstrated the importance of endogenous EFs in normal development (Levin, 2012; Smith et al., 2018; Iwasa et al., 2019; Sefton et al., 2020; Levin, 2021). Additionally, the utility of EF modulation to produce desired effects *in vitro* has been revealed, such as increased differentiation, survival or maturation of CNS cells (Figure 3) (Bertucci et al., 2019; Mobini et al., 2019; Bierman-Duquette et al., 2021; Hlavac et al., 2021). Whilst exact mechanisms of action are not understood, the beneficial effects of EF modulation have been accepted *in vivo* for multiple tissues and organs, including bone and cardiac (Leppik et al., 2018; Watson et al., 2019). Electrical stimulation-based treatments are now being implemented in the clinic, particularly for the brain (Sparing and Mottaghy, 2008; Fedorov et al., 2010; Kubis, 2016; Desmoulin-Canselier and Moutaud, 2019; Krauss et al., 2021; Sui et al., 2021).

### Applying Electrical Fields in the Brain

**FIGURE 4:**
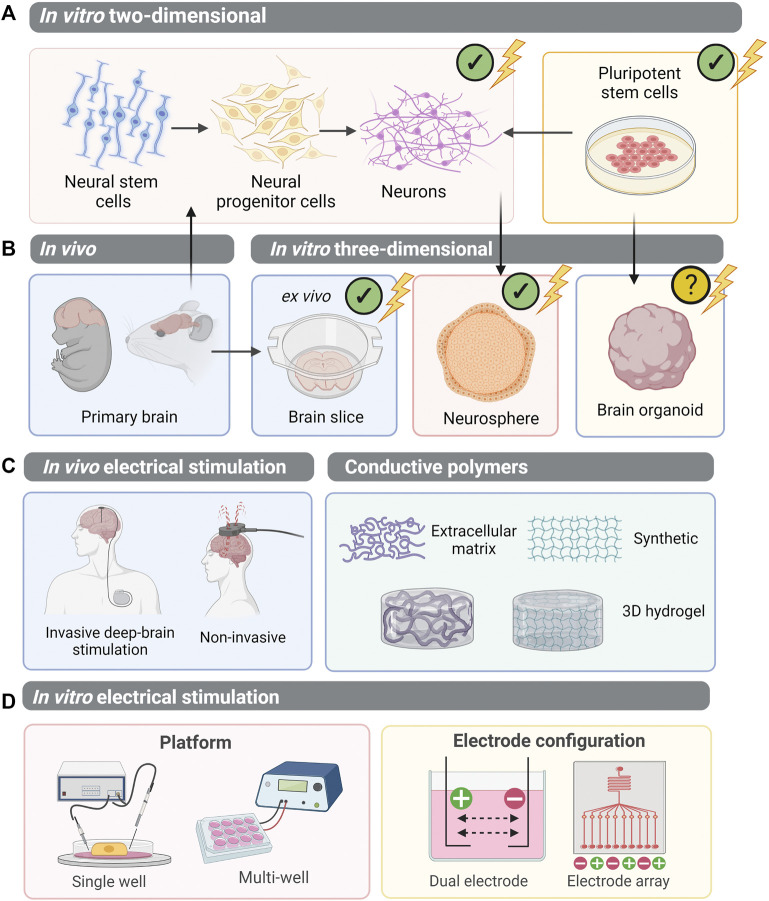
Electrical stimulation of CNS cells: moving towards PSC-derived organoids. **(A)** Multipotent neural stem cells can be isolated from the primary brain and differentiated to neural progenitor cells and neurons. Alternatively, pluripotent stem cells can be differentiated into a neural lineage. Electrical stimulation studies have been undertaken on these two-dimensional systems. **(B)** Three-dimensional systems which have been employed to study electrical stimulation *in vitro* include primary brain slices (an *ex vivo* model of the CNS), and neurosphere aggregates derived from neural progenitor cells. However, the study of electrical stimulation on pluripotent stem cell-derived brain organoids remains unexplored. **(C)** *In vivo*, electrical stimulation of the brain is performed on patients either using invasive methods, whereby electrodes directly contact brain regions, or non-invasively. To mimic *in vivo* brain stimulation in *in vitro* models of the brain, conductive polymers within 3D hydrogels have been utilised. **(D)** However, to best apply electrical stimulation to PSC-derived CNS organoids, suitable delivery platforms must be established. A single well system allows controlled delivery of electrical stimulation, whilst a multi-well plate system is higher-throughput to allow *in situ* electrical stimulation of cells. Electrodes can be configured in arrays, or pairs, generating electrical fields between each cathode (+) and anode (−). (✔), electrical stimulation experimentation performed; (?), not yet explored.

Electrical stimulation of the brain is beneficial for neurological disease, psychiatric disease and brain tumours. Non-invasive methods, including transcranial direct current stimulation (tDCS) and transcranial magnetic stimulation (TMS) have been shown to facilitate motor recovery in stroke patients (Webster et al., 2006; Fedorov et al., 2010; Kubis, 2016). Non-invasive stimulation of glioblastoma patient brains, for 20 min at a desired field strength of 0.25 V/m, altered cerebral blood flow and aided necrosis of the tumours (Sprugnoli et al., 2019). Alternatively, epidural cortical stimulation (ECS) and deep-brain stimulation, involve direct implantation of electrodes into the brain, to provide more focal electrical stimulation (Nahas et al., 2010; Balossier et al., 2015; Sui et al., 2021) (Figure 4C). This exerts desirable effects on neuroplasticity, improving neural network connectivity and topological reorganisation in cases of neurodegeneration (van Hartevelt et al., 2014). Typically, patients receiving deep-brain stimulation, such as those suffering from neurodegenerative diseases, are adults with fully formed neural structures. However, the adult brain contains a small population of neural stem cells (NSCs) arising from the subventricular zone. NSCs are self-renewing and multipotent, giving rise to neural progenitor cells (NPCs). NPCs, which have a finite replication potential, form most, if not all, of the neural and glial cells of the CNS (Morest and Silver, 2003; Merkle et al., 2004). NSCs or NPCs can be isolated from primary brain and cultured *in vitro*, both in 2D, or 3D aggregates deemed neurospheres (Figure 4A). In contrast to organoids, neurospheres lack cytoarchitecture or complex organotypic-like organisation. Nonetheless, when engineering neural organoids, we aim to replicate neurogenesis *in vitro via* guiding PSCs into NPCs, before forming more mature neural and glial subtypes. It is therefore interesting to consider the effect of EF modulation on neural stem cell, progenitor cell populations or neurospheres.

Effects of brain stimulation from the clinic have been translated to NPCs and NSCs *in vitro.* Many studies demonstrate neural cells are electrosensitive, responding with behavioural or functional changes to EFs. Applied exogenous electrical stimulation promotes survival of NSCs both *in vivo* and *in vitro* (Du et al., 2018; Sefton et al., 2020). Specifically, Sefton et al. (2020) demonstrate a significant increase in the number NPCs in neurospheres stimulated at 250 mV/mm for 3 h, formed from mouse brain-derived NPCs *in vitro*. NPCs in stimulated neurospheres were identified to have an increased capacity for survival, with an increased number of cells entering S-phase of the cell cycle. Interestingly, addition of stimulated conditioned media to non-stimulated neurospheres was not sufficient to induce this increased neurogenesis phenotype. Thus, niche-released factors were not responsible for the increase in neurogenesis, but rather intrinsic cell cycle modulation (Figure 3) (Sefton et al., 2020). In foetal NPCs derived from the mouse embryo, brief (50 μs or 200 μs) low-voltage biphasic electrical stimulation pulses significantly promoted cell proliferation (Chang et al., 2011). Similar phenotypes have been identified *in vivo*, where stimulation of the adult mammalian cortical brain *via* electrode implantation, leads to an increase in the proliferative pool of NPCs (Stone et al., 2011; Sefton et al., 2020). Targeted stimulation of the Entorhinal Cortex (EC), a region of the mouse hippocampus, resulted in increased proliferative cells in a projecting region, the denate gyrus. Remarkably, once these cells matured and integrated into hippocampal neural circuits, an increased spatial memory response, measured by water maze, was observed (Stone et al., 2011). In humans, the brains of neurodegenerative patients who received deep-brain stimulation have been examined by immunohistochemistry (Vedam-Mai et al., 2014). Compared to brains of unstimulated patients, the levels of proliferative cells in the subventricular zone, the birthplace of NPCs, were two-six fold greater (Vedam-Mai et al., 2014). From the subventricular zone *in vivo*, NPCs must also migrate long-distance, particularly to give rise to the olfactory bulb. Migration occurs along a route deemed the rostral migratory stream (Lois and Alvarez-Buylla, 1994; Curtis et al., 2007). Endogenous electrical fields, with a field strength of ∼3 mV/mm, help to guide this migratory behaviour (Cao et al., 2013). Further studies have identified endogenous EFs surrounding the cerebral cortex capable of inducing migration of transplanted NPCs (Iwasa et al., 2019). *In vitro*, application of physiologically relevant EFs to rat embryonic brain-derived NPCs facilitates migration towards the cathode (Li et al., 2008). Thus, it is postulated that NPCs respond to electrical stimulation, both *in vivo* and *in vitro* with directed migratory behaviour (Babona-Pilipos et al., 2011; Babona-Pilipos et al., 2015; Feng et al., 2017; Iwasa et al., 2019; Sefton et al., 2020). Application of exogenous EFs can alter endogenous EF-guided migrations but may not be sufficient to reverse endogenous EF effects entirely (Iwasa et al., 2019).

Collectively, studies described in this review indicate the viability of cells *in vivo* and *in vitro* in mild electrical fields conditions, typically within a range of 25-250mV/mm (Wang et al., 2013; Sefton et al., 2020). However, as with all cell-cycle related effects, manipulation of electrical fields may either promote, inhibit, or exert no effect on cell death mechanisms. This likely depends on stimulation parameters, and tissue of interest. Electrical stimulation of cells *in vitro* suggests a proportional relationship between cell death and the duration and number of electrical stimulation pulses (Matsuki et al., 2010). Furthermore, electrical stimulation at higher voltages induces formation of pores in cell membranes (electroporation) which may result in induction of apoptosis or necrosis with prolonged pulse lengths beyond the nano-second scale (Oshima et al., 1997, Oshima et al., 1998; Hofmann et al., 1999; Al-Sakere et al., 2007). Researchers have appropriately manipulated the parameters of electrical stimulation *in vivo* and *in vitro* to target aberrantly dividing malignant cells, and promote tumour ablation (Wang et al., 2012; Chen et al., 2014). However, the application of low voltage biomimetic electrical fields, below those capable of breaking the membrane, may also induce apoptosis of carcinogenic cells, specifically through increased Ca^2+^ transport (Matsuki et al., 2010). Mild (15 V, 7.5 V/mm) EF-induced Ca^2+^ flow, promotes apoptotic signalling through the activation of caspase-8 and caspase-9, upstream of caspase-3 (Matsuki et al., 2010). Promisingly, most reports of cell death-related phenotypes in response to electrical stimulation of the CNS describe a reduction in apoptosis. *In vivo* application of electrical stimulation to the cerebral cortex exerted anti-apoptotic effects in an ischemic stroke rat model, whereby ischemia-induced cell death is a major aetiology (Baba et al., 2009). The phosphoinositide 3-kinase (PI3K) pathway, a major player in cell cycle regulation, survival and apoptosis, is activated in the electrically stimulated cerebral cortex, leading to a reduction in apoptosis and ischemia-induced pathology. These findings were similarly confirmed *in vitro* using primary rat-derived NPCs from the olfactory bulb (Wang et al., 2013). Specifically, the authors examined the effects of biphasic pulsed mild EFs on growth factor-deprivation activated apoptosis. Electrical stimulation at 25 mV/mm and 50 mV/mm decreased rates of apoptosis and necrosis in NPCs cultured in serum-free conditions. Specifically, these neuroprotective effects were inhibited *via* blocking of PI3K/Akt (Wang et al., 2013). Electrical stimulation upregulated phosphorylation of Akt, and despite the absence of growth factors in culture conditions, BDNF was upregulated after continued (12 h) stimulation (Wang et al., 2013). Since PI3K signalling is a major cell cycle regulator, EF-induced mechanisms may control multiple fundamental processes, including survival, proliferation and differentiation, along with apoptosis. Similarly, growth factor BDNF contributes to neural cell survival, growth, differentiation and maturation. Therefore, multiple studies demonstrated that EF modulation could mediate distinct control of cell cycle and survival. These changes may also translate to changes in neurogenic differentiation capacity and the profile of stem cell-derived neural populations (Figure 4).

Electrical stimulation is reported in multiple studies to increase differentiation of NSCs and progenitor cells into neurons and oligodendrocytes both *in vivo* and *in vitro* (Ariza et al., 2010; Stewart et al., 2015; Chang et al., 2016; Du et al., 2018; Dong et al., 2019; Sefton et al., 2020). EFs of ∼300–400 mV/mm are reported to increase neurogenesis of *Beta tubulin III +* neurons, whilst field strengths of lower magnitudes may promote NPC proliferation and expansion in favour of differentiation (Ariza et al., 2010; Dong et al., 2019; Sefton et al., 2020; Hlavac et al., 2021). *In vivo,* hyperpolarisation of cerebral progenitor cells (radial glial) *via* over-expression of potassium channels, induced pre-emptive differentiation of typically later-born, mature neural subtypes (Vitali et al., 2018). Lineage tracing determined EF modulation *via* ion channel manipulation specifically regulated the division mode of NSCs. Notably, effects on neuronal subtype fate determination could be reversed with de-polarisation (Vitali et al., 2018). Thus, *in vivo*, modified EFs can function as fate determinants for NPCs. The increased maturation of NPCs in response to increased TEPD is a cue that could be mimicked *in vitro* to promote neurogenesis and maturation. Studies examining EF modulation of human NSC lines also identified altered fate bias. Exogenous electrical stimulation of human NSCs increased induction of neuron differentiation, as defined by a larger population of cells expressing neuron marker *MAP2* (Stewart et al., 2015; Tomaskovic-Crook et al., 2019). Within these stimulated MAP2+ neurons, an increase in neurite outgrowth was also observed (Stewart et al., 2015; Tomaskovic-Crook et al., 2019). Increased EF-induced neurite branching and length is similarly found in studies applying exogenous electrical stimulation to foetal mouse-derived and immortalised human brain derived-NPCs (Chang et al., 2011; Pires et al., 2015). Sufficient neurite branching is important for formation of functional neuronal networks, suggesting stimulation could also enhance the structure and functionality of *in vitro* CNS models. Taken together, these findings indicate a potential for EF modulation to promote cell cycle-related effects in neural cells (proliferation, survival and differentiation) to aid recapitulation of neurogenesis from PSCs.

## Pluripotent Stem Cells-Derived Cultures: What Do We Know So Far?

Surprisingly, studies translating these findings into neural differentiation of ESCs and iPSCs are very limited. Yamada et al. (2007) described the first study showing mild electrical stimulation of mouse ESCs resulted in increased propensity for neural differentiation. The ESC-derived NPCs generated from electrically stimulated cultures were reported to have a broad capacity for neuronal subtype specification, with greater plasticity than early ESC-derived 2D neuronal differentiations (Yamada et al., 2007). Furthermore, when transplanted into injured spinal cord, NPCs from stimulated mESC-derived cells were more capable of incorporating and forming neural cells than unstimulated controls (Yamada et al., 2007). Previous findings of EF-induced differentiation and fate determination in studies using primary or human NSC lines, were similarly replicated in iPSC-derived neuronal cultures (Stewart et al., 2015; Tomaskovic-Crook et al., 2019; Tomaskovic-Crook et al., 2020; Oh et al., 2021). Specifically, iPSCs were cultured in clusters in suspension within a polymer, followed by neuronal differentiation and electrical stimulation. The resultant neuronal cultures contained more MAP2+ neurons and fewer glial cells than unstimulated counterparts (Tomaskovic-Crook et al., 2020).

Since PSCs are prone to genomic and epigenetic instability, there may be hesitancy to apply exogenous stimuli which could adversely alter pluripotent capacity. However, exogenously stimulated human ESCs and iPSCs have been demonstrated to give rise to all three germ layers, with accelerated or enhanced differentiation capacity (Yamada et al., 2007; Tomaskovic-Crook et al., 2020). *In vivo,* endogenous EFs are present at stages overlapping with the division of pluripotent embryonic stem cells (Levin et al., 2002). Therefore, EF modulation has the potential to aid morphogenesis from the earliest stage *in vitro* and effects of EF-modulation prior to the NPC stage should not be overlooked. Further studies on electrical stimulation of PSCs and ESCs are required to understand EF-induced fate determination, which will aid optimisation of PSC differentiation protocols.

## Enhancing Organoid Cultures With Bioelectricity

### A Potential Tool for Next Generation Central Nervous System Organoids

**FIGURE 5:**
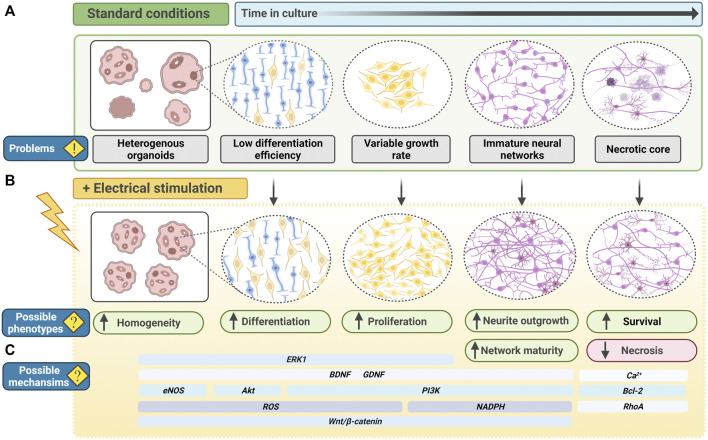
Electrical stimulation to improve PSC-derived CNS organoids. **(A)** In current standard culture conditions, PSC-derived organoid systems, for example neural organoids, have several problems or shortcomings. Organoids may be heterogeneous and exhibit inter-differentiation or inter-cell line variability. Neural stem cells may have a low differentiation efficiency, and neural progenitor cells may have a variable growth rate. With increased time in culture, neural networks begin to form but neurite outgrowth and branching may be minimal, limiting functionality. As organoids continue to grow, oxygen transport to the centre of the organoid is limited, leading to the formation of a necrotic core. **(B)** Electrical stimulation of neural organoids may enhance phenotypes relating to the current problems in organoid cultures. Functioning as an additional physiological cue, electrical field modulation may reduce heterogeneity in differentiation and enhance neurogenesis. Neural stem cells and progenitor cells may have an increased capacity for differentiation and proliferation. Electrical fields may also promote neurite outgrowth and branching, creating more mature neural networks with more potential for functionality. Electrical fields may also prevent apoptosis and enhance cell survival, decreasing the formation of necrotic core in older organoids. **(C)** Many potential molecular mechanisms are implicated to respond to electrical field modulation, including transient oxidative or ionic signals, and canonical signaling networks capable of regulating a wide-range of cell cycle-related and metabolic processes. Electrical stimulation-induced signals may therefore act throughout organoid development to induce phenotypes that could improve organoid development, differentiation and maturation. BDNF, Brain derived neurotrophic factor; GDNF, glial cell line–derived neurotrophic factor; PI3K, phosphoinositide 3-kinase; ROS, reactive oxygen species.

In summary of the above, EFs are capable of enhancing neurogenesis both *in vitro* and *in vivo*. Despite the overwhelming evidence that endogenous electrical stimulation promotes neurogenesis, researchers are yet to explore the potential of bioelectric cues in guiding PSC-derived CNS organoids. The proof-of-principle studies on mouse and human iPSCs and ESCs support the ability of electrical stimulation to be employed in neural cultures at the PSC stage without hampering differentiation capacity (Yamada et al., 2007; Tomaskovic-Crook et al., 2020). The biological processes regulated by EFs in NSC and NPC cultures, align closely with the processes involved in generating, and optimising, PSC-derived organoids. This includes increases in survival, proliferation, differentiation and maturation of neural cells (Figures 3, 5).

PSC-derived brain organoids represent one of the most advanced *in vitro* human PSC-derived models. However, several unpredictable variables continue to arise in culture. For example, in the case of unguided organoids, reliance on self-patterned differentiation results in variability from batch-to-batch differentiation and heterogeneity of organoids (Figure 5A). Non-neuroectodermal cells are also identifiable in cerebral organoids (Quadrato et al., 2017). Given that studies unanimously report EF modulation to have an inducing and enhancing effect on neurogenesis, exploiting EFs in organoid cultures could be a legitimate approach to improving the success rate of neurogenesis and reducing differentiation heterogeneity (Figure 5B). Electrical stimulation of ESCs *in vitro* is reported to bias fate towards NPCs (Yamada et al., 2007). Furthermore, PSCs stimulated at early phases of brain organoid differentiation may offer more robust induction of neuroectodermal, and subsequent neural lineage cells.

Guided protocols offer less batch-to-batch variability. However, the addition of extrinsic factors does not account for existing endogenous levels of molecular cues present in PSCs. PSC lines vary intrinsically and therefore each new protocol needs to be optimised to generate the desirable cytoarchitecture (Strano et al., 2020). This is also a more artificial style of differentiation, which may be suitable for high-throughput studies such as drug screening, but less appropriate for studies of development or fate determination. EF modulation is reported to upregulate neurotrophic factors, particularly in a 3D environment, therefore electrical stimulation of brain organoids could endogenously upregulate key neural-determinant signalling molecules (Figure 5C) (Song et al., 2019). This would offer a valuable opportunity to reduce addition of extrinsic factors in organoid differentiation, both limiting interference in the intrinsic differentiation process and associated costs for laboratories.

In the case of cerebral organoids with multiple regions, the 3D tissues grow to a considerable size, up to 4 mm in diameter, and transport of oxygen and nutrients becomes limited, restricting overall organoid growth and maturation (Sutcliffe and Lancaster, 2019). To counteract this, cultures are maintained on spinning bioreactors or shakers to improve oxygen diffusion in the media and thereby organoid survival (Lancaster et al., 2013; Lancaster and Knoblich, 2014; Qian et al., 2016). However, EF-induced effects on increased cell survival and reduced cell death hold promise for additional implementation in organoid cultures.

To enhance brain organoids functionality, the formation of mature neural networks is key. Both primary and PSC-derived neural cells respond to electrical stimulation with increased neurite outgrowth (Fields et al., 1990; Chang et al., 2011; Pires et al., 2015; Stewart et al., 2015; Tomaskovic-Crook et al., 2019). Electrical stimulation is able to rescue stunted neurite growth in neuregulin-1 knockout primary-derived cortical cells (Zhang et al., 2018). Moreover, when comparing electrical stimulation in a 2D versus 3D environment, whilst 2D cells exhibited increased neurite outgrowth from individual cells, in 3D cells formed aggregates which in turn formed neurite bundles (Zhang et al., 2018). These projected into neighbouring cell aggregates, creating more complex and dense neural networks that better resemble the *in vivo* brain (Zhang et al., 2018). Neurite density is tightly linked to pathology in psychiatric, neurodegenerative and neurological disease (Saad et al., 2015; Grussu et al., 2017; Kamagata et al., 2017; Rae et al., 2017; Song et al., 2018; Wang et al., 2019; Prem et al., 2020). The utility of EF modulation to increase neurite outgrowth and neural network density therefore provides a wealth of opportunity to model disease, therapeutics and enhanced functionality of brain organoids *in vitro*.

Retinal organoids have also been a thriving area of research. Diseases of the retina, such as inherited retinal diseases, or age-related macular degeneration are leading causes of blindness. Similarly to neurodegenerative diseases of the brain, retinal degenerations are amenable to therapeutic EF modulation. Electrical stimulation has been applied to the retina *via* several modes *in vivo*, either targeting the retina from the front or side of the eye, non-invasively, or alternatively directly contacting the retinal tissue (sub-retinal). Non-invasive EF modulation has been demonstrated to preserve vision in diseased retina in both animal models and clinical trials (Reviewed in Sehic et al., 2016; Sanie-Jahromi et al., 2021). As with deep-brain stimulation, further studies are required to elucidate full mechanisms of action. The human retina differs significantly from that of lower order animals, and human PSC-derived organoids therefore provide a better platform to study EF-induced therapeutic effects on the human retina. *In vitro*, primary eye-derived retinal glial cells (Müller Glia) were found to respond to electrical stimulation with increased proliferation and expression of cell fate determinant genes (Enayati et al., 2020). Specifically, this was linked to the action of Calcium signalling (Enayati et al., 2020). Several studies have also reported a regenerative response of the output neurons of the retina, the retinal ganglion cells, in response to biomimetic stimulation (Fu et al., 2015; Gordon, 2016). However, the retina contains multiple cell types which are likely to have differing responses to stimulation. Thereby, testing of stimulation within PSC-derived organoids that contain multiple retinal cell subtypes would allow better characterisation of cell-specific phenotypes.

Spinal cord injuries have a devastating impact on quality of life with a huge global disease burden. Therefore, regenerative medicine approaches, or modelling of diseased spinal cords is a compelling objective. Currently, there is no cure for spinal cord damage and injuries are irreversible. The spinal cord contains a niche of NSCs which do not demonstrate multipotency or undergo neurogenesis *in vivo* (Johansson et al., 1999; Horner et al., 2000; Shihabuddin et al., 2000). However, when isolated and injected into the brain, spinal cord derived-NSCs are capable of producing neurons (Shihabuddin et al., 2000). This led to the hypothesis that the spinal cord microenvironment is masking the stemness of these cells (Becker et al., 2018). Considering the established role of EFs in modifying the tissue microenvironment to induce neurogenesis, electrical stimulation of spinal cord tissue holds great promise. *In vivo*, clinical application of electrical stimulation for spinal cord injuries has been reported to restore motor activity in paralysed patients (Reviewed in Marquez-Chin and Popovic, 2020; Karamian et al., 2022). Primary spinal cord-derived NSCs, alike to brain-derived NSCs, respond to EFs with directed migratory behaviour (Babona-Pilipos et al., 2011; Meng et al., 2012). This could be harnessed *in vivo*, to activate and recruit NSCs to the area requiring regeneration. Moreover, electrical stimulation holds the potential to modulate neuroinflammation, which is a major aetiology of neuronal death following spinal cord injury (Rust and Kaiser, 2017; Hellenbrand et al., 2021). Application of EFs *in vivo* is demonstrated to reduce inflammatory response in the CNS, partly *via* microglia deactivation (Ayanwuyi et al., 2021; Park et al., 2021). However, studies of the effects of electrical stimulation on 3D spinal cord injury models are minimal. Meng et al. (2012) describe a protocol to compare EF-induced effects on NPC migration in both a 2D format and 3D *ex vivo* spinal cord slice platform. Organ slice models present an *in vitro* system with a microenvironment more representative of *in vivo*. However, PSC-derived organoids would provide an unlimited resource of spinal cord tissue for study of the complex mechanisms of EF on this unique population of NPCs. Recently, an organ-on-a-chip model using hPSC-derived spinal cord organoids demonstrated electrophysiological function *via* microelectrode array (MEA) (Ao et al., 2022). Electrical stimulation could easily be implemented in this system to observe neurogenic responses, whilst co-culture with endothelial cells could probe effects on neuroinflammation.

### Moving to the Third Dimension: Interfacing With Biomaterials

As researchers in the field have recognised the limitations of 2D-based *in vitro* models, studies have focused on cultures of NPCs in a 2D or neurosphere format, mediated with biomaterials and scaffolds (Figure 4). Bioengineered polymers and scaffolds represent a unique opportunity to explore electrical field modulation *in vitro*, due to the ability to confer electro-conductive properties on polymer formulations. Organic polymers can be “doped” with anionic biomolecules to confer innate conductivity and biocompatibility. These electroconductive polymers, such as polypyrrole (PPy) and poly(3,4-ethylenedioxythiophene) (PEDOT) are readily manufactured with bioprinting. An increasing number of studies utilise conductive polymers to deliver electrical stimulation to NSCs and NPCs *in vitro* for tissue engineering purposes (Reviewed in Bierman-Duquette et al., 2021). In fact, culturing of NPCs on conductive polymers alone, without exogenous stimulation, may enhance the survival and proliferation, polarisation and axonal projections, or differentiation and maturation of NPCs (Luo et al., 2013; Shin et al., 2017; Wang et al., 2018; Garrudo et al., 2019). Promisingly, this has also been linked to improved electrophysiological functionality (Shin et al., 2017). When compared to bare hydrogels, the addition of PPy or carbon nanotube electroconductive motifs in 3D hydrogel-cultured NPCs caused an increase of calcium channel expression, depolarisation and intracellular calcium influx (Shin et al., 2017). Thus, providing an electroconductive environment alone for NPCs may promote endogenously-driven EF-mediated effects.

Combined with exogenous electrical stimulation, studies report the use of conductive polymer 2D-based platforms to enhance or accelerate neurogenesis, differentiation and neural maturation (Li et al., 2013a; Stewart et al., 2015; Kwon et al., 2021; Sordini et al., 2021). Transcriptomic analysis of PPy-stimulated human NPCs, originally derived from ESCs, identified differential expression of multiple genes implicated in survival and synaptic modelling, and the VEGF-A pathway which plays major roles in the cell cycle and neuroprotection (Georges et al., 2006). Advantageously, the use of biomaterials in *in vitro* cultures has facilitated transformation from 2D-based electrical stimulation to 3D-based studies by utilising biomaterial-based scaffolds (Reviewed in Bertucci et al., 2019). 3D biomaterial-mediated cultures of both brain-derived and iPSC-derived NPCs have been demonstrated to be numerously advantageous to 2D, increasing cell survival, proliferation and maturation (Cukierman et al., 2001; Georges et al., 2006; Banerjee et al., 2009; Chandrasekaran et al., 2017; Mauri et al., 2018). Therefore, emerging studies are now incorporating 3D-based conductive polymer electrical stimulation platforms for NPC culture (Li et al., 2013a; George et al., 2017; Xu et al., 2018; Zhu et al., 2018; Song et al., 2019; Tomaskovic-Crook et al., 2019). Direct comparison of primary mouse NSCs receiving stimulation on a 2D or 3D graphene-based foam identified increased proliferation and differentiation in the 3D-cultured NSCs (Li et al., 2013a). Human brain-derived NSCs, and then later colonies of iPCS, have been cultured and electrically stimulated in a 3D environment *via* encapsulation with PEDOT-based hydrogels. (Tomaskovic-Crook et al., 2019; Tomaskovic-Crook et al., 2020). In the encapsulated environment, both the iPSCs and NSC formed clusters and differentiated into neurons. Culturing of the cells in the 3D environment re-affirmed findings previously established in 2D cultures of human brain-derived NSCs (Stewart et al., 2015). Specifically, in both 2D and 3D formats for NSCs or iPSCs, conductive polymer-mediated electrical stimulation resulted in increased neural fate induction, as demonstrated by increased expression of Beta-III-Actin + neurons (Stewart et al., 2015; Tomaskovic-Crook et al., 2019; Tomaskovic-Crook et al., 2020). In addition, the resultant neurons displayed longer and more branched neurites. However, the ability to study this phenotype in a 3D environment, and better examine the network of neurite extensions within the 3D micro-environment, is far more informative. Interestingly, Song et al. (2019) compared electrical stimulation of hiPSC-derived NPCs using a 2D and 3D platform. Using NPCs embedded in a 2D PPy hydrogel film or 3D PPy tube structures, the authors demonstrate the relation of dimensionality to EF-induced effects. In both a 2D and 3D setting, stimulated NPCs showed differential expression in neurotrophic factors when compared to controls, including heparin binding EGF like growth factor (HBEGF), heat shock protein family member 1 (HSPB1), glial cell derived neurotrophic factor (GDNF), brain derived neurotrophic factor (BDNF), and neurotrophin 3 (NTF3). However, there were significant differences between the two. For example, GDNF and BDNF were far more upregulated in the 3D platform (Song et al., 2019). These are important factors regulating brain development, differentiation, survival and maturation (Allen et al., 2013; Popova et al., 2017). Accordingly, BDNF and GDNF have been implemented *in vitro* to guide differentiation of brain organoids, including region-specific forebrain (Birey et al., 2017; Qian et al., 2018), midbrain (Kim et al., 2019), cerebellar (Holmes and Heine, 2017), telencephalic (Mariani et al., 2015, 1), as well as cerebral organoids (Quadrato et al., 2017; Watanabe et al., 2017). Specifically, addition of GDNF and BDNF is shown to enhance the maturation of neuronal networks and improve electrophysiological function in forebrain organoids (Qian et al., 2018).

Apart from improved development and maturation for modelling *in vitro*, another major utility of PSC-derived stimulated cells is for regenerative medicine. Promisingly, ESC-derived and stimulated NPCs cultured on a PPy scaffold were more able to contribute to neural regeneration following implantation in a stroke-model rat brain, than unstimulated NPCs (George et al., 2017). However, for the purpose of better mimicking *in vivo* morphology and function, organoids represent a far more advanced *in vitro* model than neurospheres or NPCs, and thus provide a more robust source of cells or tissue for transplantation.

### Bridging the Gap in Bioengineering: Electrical Stimulation of Organoids

In the move towards “next-generation” organoids, techniques such as micro-patterning of morphogen gradients, 3D bioprinting of substrates and microfluidic devices are being used to improve organoid cultures of various tissues, including lung, kidney and intestine (Reviewed in Garreta et al., 2021; Yi et al., 2021). For brain organoid differentiation, Matrigel was early implemented to aid biomechanical development. Commercially available decellularized matrices, such as Matrigel, are the most easily attained biomaterials for cell biology laboratories (McCrary et al., 2020). These matrices contain cell-derived ECM proteins along with biochemical factors that typically have wide-spread effects on the cell cycle, such as FGF and EGF (Vukicevic et al., 1992; Kleinman and Martin, 2005). However, concentrations of growth factors are unknown and undefined proteins may also be present, which may contribute to batch-to-batch heterogeneity in organoid differentiation. Whilst growth-factor reduced variants are now available, a chemically defined synthetic polymer composition offers an alternative approach to reduce this issue. Currently, few biopolymers have been explored to support PSC-derived brain organoids. Culturing brain organoids with a silk-fibroin based biopolymer offered tuneable biomechanical properties to aid 3D cytoarchitecture formation (Tang-Schomer et al., 2014). In another study, a calcium-alginate hydrogel, formed into hollow fibres, was combined with a microfluidic device to form brain organoids from iPSCs (Zhu et al., 2017). Thus, the device provided a physically controlled environment to sustain organoid development, with the ability to exchange oxygen and nutrients using microfluidics (Zhu et al., 2017). An alternative to Matrigel is a chemically defined hyaluronan-based hydrogel which has been proven to support induction of human iPSCs into cerebral organoids, but not in long-term culture (Lindborg et al., 2016). Altering the properties of Matrigel, by tuning with an alginate polymer, has been explored for embedding of brain organoids (Cassel de Camps et al., 2022). Encapsulating brain organoids in alginate-tuned Matrigel, which increases stiffness, skewed cell populations towards a more mature neural fate, although in a trade-off for growth capacity. Here, the authors describe differing morphology of brain organoids in response to differing matrix stiffness (Cassel de Camps et al., 2022). This stresses the importance of the biomechanical microenvironment on the self-organisation program of PSC-derived neural organoids. Successful implementation of biopolymers that provide suitable culturing conditions for 3D PSC-derived brain organoids will require optimisation. To date, brain organoids have not yet been combined with conductive polymers such as PPy or PEDOT. However, the addition of alginate or calcium to hydrogels likely confers increased conductive properties, and electrical stimuli could more readily be applied to these formulations which have already been tested in brain organoid differentiations (Kaklamani et al., 2018; Zhu et al., 2018; Cassel de Camps et al., 2022).

### Can We Generate Complex Organoids Using Electrical Bio-Modulation?

Exploiting EF modulation *via* conductive polymers in brain organoids present several exciting opportunities to generate more advanced *in vitro* models of the brain and CNS. Markedly, brain-region specific organoid systems don’t offer the opportunity to model multiple regions and interactions between them. To address this, different brain-region organoids have been co-cultured or fused together in “assembloid” approaches (Bagley et al., 2017; Birey et al., 2017; Xiang et al., 2017; Sloan et al., 2018; Xiang et al., 2019; Miura et al., 2022). This can be achieved *via* several methods, including stepwise assembly of already formed organoids according to neurodevelopmental trajectory, or spontaneous fusion on a shaker. The fused organoids are demonstrated to functionally integrate and have transcriptomes representative of the foetal brain (Xiang et al., 2017; Miura et al., 2022). Application of exogenous EFs during the assembly process could enhance migratory behaviour between regions and promote regional fate determination. This would allow co-culturing at earlier timepoints in development to model early neurodevelopment. However, this is a forced co-culture, rather than spontaneous development from PSC stage. Ideally, to better mimic *in vivo* CNS development, brain, retina, spinal cord, and olfactory organoids would be generated spontaneously in the same dish together. Currently, the generation of multiple cell types of CNS origin may be achieved with the same media formulations, or within a single structure. For example, cerebral brain organoids contain retinal cells (Lancaster et al., 2013; Quadrato et al., 2017). Moreover, optimisation of culture conditions for cerebral organoids demonstrated development of bilateral optic vesicle structures (Gabriel et al., 2021). These were resemblant of the early embryonic retina in gene expression patterns (Gabriel et al., 2021). Forebrain structures can also spontaneously arise in PSC-derived retinal organoids cultures (Meyer et al., 2011; Ohlemacher et al., 2015) and retinal-brain assembloids have been generated (Fligor et al., 2021). Therefore, suitable biochemical signals exist to substantiate development of both brain and retina, and development of optimised biomechanical and bioelectric signals could enhance more robust multi-organ organoid formation. Additionally, both brain region and whole-brain organoids lack broader structure, particularly the characteristic cortical folding, or gyrification, present in the human brain *in vivo*. Recently, a biomaterial scaffold was used to induce a flattened morphology in brain organoids (Rothenbücher et al., 2021). The resulting organoids were size and shape controlled due to a larger surface area for oxygenation, and primitive cortical folding. This could be a beneficial prospect for high throughput screening. However, these manipulations may sway development from the innate development trajectory we are trying to recapitulate in the dish.

*In vivo*, changes in bioelectric signalling of the brain can cause major structural changes (Smith et al., 2018). The mutation of a voltage-gated sodium channel, SCN3A, causes misfolding of the human cortical brain. Mutated SCN3A causes aberrant ionic current flow during early stages of neurodevelopment (Smith et al., 2018). Therefore, the effect of exogenous bioelectricity application alone to alter organoid structure should not be overlooked.

Another major goal in neural organoid engineering is overcoming the lack of blood supply. For brain organoids, efforts have been made to create vascularisation, *via* co-culture with endothelial cells, genetic manipulation to express endothelial cell transcription factor, human ETS variant 2 (ETV2), and transplantation of brain organoids into animals to permit *in vivo* vascularisation (Mansour et al., 2018; Pham et al., 2018; Cakir et al., 2019; Shi et al., 2020). PSC-derived organoid models of the blood-brain-barrier have also been established (Bergmann et al., 2018). Bioelectric signals are reported to influence angiogenesis, the formation of blood vessels within existing vasculature. Electric field-induced angiogenesis has been identified in endothelial cells receiving electrical stimulation (Wei et al., 2020). Blood flow increases in response to electrical fields, and blood flow alone causes a change in electric field potential (Petrofsky et al., 2007; Trivedi et al., 2013). Endothelial progenitor cells, which form vasculature tissue, respond to *in vitro* application of exogenous EFs with directional migration (Zhao et al., 2012). Moreover, signalling *via* vascular endothelial growth factor (VEGF), the master regulator of angiogenesis, was found to be upregulated by EF modulation (Zhao et al., 2012). The ability of bioelectric signals to guide neo-angiogenesis is also consistent with the established role of bioelectricity in wound healing. It may therefore be a worthwhile approach to test EF-induced effects on blood vessel formation within vascularised organoids.

The work described here highlights the likely complex interplay of bioelectric signals with biomechanical and biochemical development. Importantly, a major stall in the development of more advanced multi-region or organ CNS organoids is the requirement each tissue has for a specific microenvironment, including biochemical, biomechanical, and likely bioelectrical. Thus, an ultimate approach to generating CNS organoids would utilise multiple microenvironments in the dish. For example, providing regionalised condition suitable signalling factors, and uniquely tailored biomaterials for each tissue, with the application of bioelectricity to enhance endogenously-driven mechanisms of proliferation, migration, neurogenesis and maturation.

## FUTURE WORKS

### Developing Electrical Stimulation Platforms for Organoids

Optimisation of polymers and devices to enable organoid development and electrical stimulation needs to be tailored, taking experimental requirements into consideration. Many electrical stimulation devices and conductive polymers are not readily amenable to 3D culturing conditions. For example, varying polymer stiffness and dopant ratio has direct effects on NPC survival and proliferation (Ma et al., 2016; Garrudo et al., 2019). This may limit their applications in the field of cell biology and developmental biology. Culture surface area tends to be very limited, and rigid structures of scaffolds may interrupt the native biomechanical rules of organogenesis. One of the allures of PSC-derived brain organoid differentiation for cell and developmental biologists is the intrinsic patterning and organisation abilities. It would therefore also be worthwhile to consider the development of novel mechanisms to deliver EFs to 3D organoid cultures *in situ*, including without the use of advanced biomaterials and conductive polymers.

Establishment of suitable delivery platforms for organoids and 3D culture environments will enhance the utility of this technology and facilitate the use of stimulation devices for cell biology laboratories. Electrical charges can be delivered to cells using either direct stimulation, with conductive metal electrodes submerged in culture medium, or salt bridges, such as agar embedded in a glass capillary. This can be facilitated in any device suitable for cell culture. Both microfluidic devices, and multi-well plate electrical stimulation platforms have been developed, with the latter being more suitable for long-term organoid cultures (Figure 4D). Tomaskovic-Crook et al. (2019) developed a microelectrode array-based culture-stimulation platform, with vertical “pilar” electrodes, allowing for more high-throughput screening and generation of 3D neural tissue (Tomaskovic-Crook et al., 2019). Vertical electrodes have also been employed in a custom designed 96-well platform electrical stimulation plate, capable of providing several stimulation parameters instantaneously, permitting high-throughput screening of stimulated cells (Du et al., 2018). This design could aid the optimisation of suitable stimulation parameters for different tissues or developmental stages. Moreover, a pillar, or column-shaped vertical electrode may permit contact with organoid tissue whilst allowing maximal capacity for culture medium. Such approaches have greater utility in advancing guided differentiation protocols, whereby physical interference with cultures is of less concern. A microelectrode array design, with multiple electrodes in each well capable of generating an electric current, could also be utilised to have more precise control over the EFs generated (Figure 3D). This could probe discrete regions of an organoid, or assembloid, to promote EF-induced effects at particular timepoints according to developmental stage. On the other hand, a simpler electrode circuit design in a standard 6, 12, or 24-well plate format would allow for easier implementation, catering for organoids of varying size and media volume requirements. For example, attachment of electrodes into a plate lid for direct immersion into cell culture media may offer maximal compatibility with *in situ* suspension culture (Mobini et al., 2016; Mobini et al., 2017). Unguided differentiation protocols may favour a stimulation device with minimal interference to structural composition of the organoid, delivering stimulation directly into the media to best utilise the ions and molecules present. For fabrication of electrodes, platinum offers excellent conductivity and charge injection capacity with minimal corrosive properties (Kim et al., 2007; Xiong et al., 2015; Mobini et al., 2016; Mobini et al., 2017; Leppik et al., 2019). Careful consideration should also be given to the uniformity of EF stimulation in order to generate reproducible data. Whilst cell culture plates are typically circular, many electrode configurations generate a rectangle or square-shaped EF. One study aimed to address this, by 3D printing a polymer into a circular insert to provide a uniform EF (Tsai et al., 2016). In addition, computational modelling of EFs *in vitro* will be required, specifically to understand electrical current distribution in the 3D organoid environment and with varying media compositions. (Zimmermann et al., 2021). Ultimately, the choice of electrode material and electrochemical characterisation of the set up will be critical to determine a biologically safe stimulation regime, which delivers the required amount of charge without splitting the water or producing toxic faradaic by-products (Boehler et al., 2020; Mobini et al., 2022).

Recently, a novel 6-well plate device was generated using 3D printing to sustain combinatorial mechanical and electrical stimulation (Cortes et al., 2020). This device was optimised to consider tissue culture conditions, with FDA-approved autoclavable materials able to sustain high humidity incubator environments. Vacuum-controlled flexible membranes provided a programmable mechanic stimulation alongside electrical pulses (Cortes et al., 2020). Whilst this was used to demonstrate improved iPSC-derived cardiac differentiation, a device providing multi-modal stimulation could be similarly implemented for culture of CNS tissues to provide suitable biomechanical cues for *in vitro* organotypic culture. In another interesting example, *in vitro* electrical stimulation combined with heat-shock treatment, at 42°C, induces ESC differentiation into definitive endoderm (Koga et al., 2017).

Ideally, the prospect of delivering multiple forms of stimuli, such as thermal, mechanical, optical and electrical, suitably optimised for your tissue of interest, would be an ultimate goal in bioengineering “next-generation” CNS organoids.

### The Bottleneck: Mapping the Bioelectric Network

Altogether, these studies provide proof-of-concept that devices can be customised for specific applications and tissue culture setups. Regardless, the optimisation of parameters, including signal shape, current, frequency and duration, is a large tissue-specific task which may present a bottleneck. Pre-clinical and clinical studies have indicated parameters capable of generating therapeutic effects in a safe manner (Merrill et al., 2005; Boehler et al., 2020; Krauss et al., 2021; Neudorfer et al., 2021). For example, for deep-brain stimulation, a square shape signal, with biphasic pulses (with an anodal and cathodal phase), seems most suitable (Reviewed in Krauss et al., 2021). Biphasic stimulation is charge-balanced and may also be preferable for *in vitro* application to NPCs, due to minimising the build-up of oxidation by-products (Iwasa et al., 2019). Secondly, *in vivo* physiological measurements of EFs in cells and systems of interest should also guide stimulation paradigms. Thirdly, computational modelling and experimental measuring of stimulation experiments should be undertaken. Several analytical or theoretical methods have been developed to aid the calculation of EFs generated by different delivery platforms within a range of culture conditions (Schopf et al., 2016; Abasi et al., 2020; Boehler et al., 2020; Guette-Marquet et al., 2021; Zimmermann et al., 2021). One study aimed to address this by developing a “digital twin” of an electrical stimulation device (Zimmermann et al., 2021). This is a digital representation of the device, (a computational model) which is also able to compute a real set of data, and accordingly respond or adapt the model based on the data (i.e., machine learning). This provides the ability to measure complex electrochemical reactions at the electrode-electrolyte interface. The capability to record or predict the movement of EFs *in vitro* would be ideal for applications to organoid cultures, where the complexity of electrical current patterns is likely enhanced by the additional dimensionality of 3D cultures. Alternatively, to offer real-time measurements of experimental conditions, another study developed a dual-function electrical stimulation and recording apparatus. This monitors the electrochemical status of the culture conditions using impedance spectroscopy (Abasi et al., 2020).

To understand the relevance of these electrochemical conditions caused by exogenous electrical stimulation, we need a more detailed understanding of endogenous EFs in the human CNS. *In vivo*, EFs are present at the cellular level, at the level of the whole epithelium or tissue, and even the whole-organism or embryo, mediating development or regeneration (Levin et al., 2017). Mapping of bioelectric networks at each level is therefore a highly complex task. Studies of developmental bioelectricity are working to unveil these networks in small model organisms (Levin, 2014; Levin et al., 2017; Levin, 2019; Levin, 2021). Molecular mechanisms of EF-induced effects have been revealed by studying the proteome and transcriptome, as genomic regulation is powerfully connected to the bioelectric state. Through the expression of ion channels and regulators, bioelectric state can be genetically programmed. Correspondingly, through the downstream activation of transcription factors, bioelectric states may drive gene expression. However, the same bioelectric state of a cell may be achieved using various mechanisms. A multitude of ionic combinations or changes in transporter permeability may confer the same overall net effect on cellular charge and TEP. Therefore, multi-omics alone will not capture the complexity of bioelectric networks, and this data will require integration with both *in silico* computational modelling, and physiological measurements of EFs.

Once we better understand these studies we can begin to ask: to what extent are we recapitulating these endogenous electrical fields *in vitro* in our PSC-derived organoids?

### Molecular Bioelectricity: the Interplay of Electrical Stimulation and Endogenous Signalling

Endogenous electric fields, variations in TEP and exogenous application of electrical stimulation have been linked to multiple canonical signalling networks and metabolic pathways (Figure 5C). In particular, EFs have been identified to regulate pathways involving phosphatase and kinase activity. Numerous reports have confirmed the ability of EFs to induce activation of Akt, and signal transduction of the PI3K pathway (Arocena et al., 2010; Meng et al., 2011; Wang et al., 2013; Dong et al., 2019; Yang et al., 2019; Wei et al., 2020). Additionally, multiple studies have identified various electrical field parameters to increase production of nitric oxide (NO) both *in vivo* and *in vitro* (Petrofsky et al., 2007; Kim et al., 2013; Trivedi et al., 2013; Wei et al., 2020). Specifically, EFs induce activating phosphorylation of eNOS, and levels of phosphorylation are proportional to electric field potential (Wei et al., 2020). PI3K inhibitors prevent EF-induced eNOS and Akt activation (Wei et al., 2020). In neuronal cells, PI3K/Akt signalling mediates EF-induced differentiation, dependent on expression of Achaete-scute homolog 1 (Ascl1) (Dong et al., 2019). In spinal cord injured rats, electrical stimulation was identified to promote neural survival through RhoA signalling and ERK1/2-Bcl-2 pathway (Joo et al., 2018). Phenotypically, increased RhoA signalling and ERK1 signalling results in increased neurite outgrowth and decreased apoptosis in the spinal cord of electrically stimulated rats (Joo et al., 2018). Other studies implicate Wnt signalling as a target for EF modulation, for example by promoting the migration and neurogenesis in EF-modulated rat brains through Wnt/GSK3β (Liu et al., 2013). Genetic manipulation of ion channels also determined EF-induced neuronal maturation *in vivo* to involve inhibition of canonical Wnt/beta-catenin signalling (Vitali et al., 2018). However, another study found Wnt signalling alone was not sufficient to induce EF-specific effects on neural progenitor proliferation (Sefton et al., 2020).

Other, more transient signalling molecules may also function as downstream bioelectric signals. For example, the upregulation of calcium signalling has also been directly implicated in EF-induced neural fate determination (Yamada et al., 2007; Shin et al., 2017; Enayati et al., 2020). Reactive oxygen species (ROS), such as hydrogen peroxide (H_2_O_2_) are ubiquitous signalling molecules with defined roles in axial patterning, CNS development, differentiation and regeneration (Reviewed in Coffman and Su, 2019). NADPH oxidase, which catalyses H_2_O_2_ production, can be activated by exogenous electrical currents (Chatterjee et al., 2012; Li et al., 2013b). During amphibian regeneration, H_2_O_2_ and V_mem_ depolarisation overlap spatiotemporally. Studies using NADPH oxidase inhibitors during *Xenopus* regeneration suggest a two-way regulation mechanism intertwining redox reactions and electrical fields (Ferreira et al., 2016). The authors postulate that a change in membrane potential provides a rapid and dynamic signal for NADPH activation. Spatiotemporal H_2_O_2_ gradients exist throughout development and may therefore provide a blueprint for bioelectrical activity. Primary embryo-derived NSCs exhibit higher levels of mitochondrial and cytoplasmic ROS than committed NPCs (Khacho et al., 2016). This is mediated by the changing mitochondrial dynamics as NSCs commit to progenitor stage. Thus, increased ROS-mediated signalling functions to supress self-renewal and promote differentiation *via* Nuclear factor-erythroid factor 2-related factor 2 (Nrf2) signalling (Khacho et al., 2016). Accordingly, endogenous ROS signalling is important for the reprogramming of somatic cells to iPSCs*,* and ROS levels are stringently controlled during growth of iPSCs and ESCs *in vitro*, along with NSCs (Lee et al., 2012; Yang et al., 2013; Yeo et al., 2013). As such, any effect of exogenous electrical fields on ROS production in PSC-derived cultures should be monitored, as stage-specific control of ROS production may be desirable. Build-up of by-products in PSC and organoid cultures should also be mitigated with immediate and frequent changing of culture medium after electrical stimulation.

A detailed review of bioelectric-driven molecular mechanisms governing development has recently been produced (George and Bates, 2022), and is beyond the scope of this review. However, the ability of EFs to influence canonical signal transduction and metabolic programming therefore provides a wealth of mechanisms by which electrical stimulation may enhance cell survival, migration, proliferation and differentiation of PSC-derived organoid cultures (Figure 5). Moreover, PSC-derived organoids provide micro-physiological systems that are amenable to EF manipulation *in vitro*, to aid delineation of molecular mechanisms directing EF-induced phenotypes. Downstream multi-omics analysis, including transcriptomics, proteomics and metabolomics, combined with functional analysis, such as electrophysiology will be key in identifying targets of bioelectricity and correlating this with phenotypic response. Advantageously, some EF-induced signals, such as ROS and Ca^2+^ may be rapidly and dynamically generated in response to electrical stimulation, allowing stage-specific control over PSC differentiation and organoid development.

### Translational Relevance: Disease Modelling and Electrotherapy

Disease modelling and development of novel therapeutics are a major utility of CNS organoids. As a standalone therapy, electrical stimulation of the CNS is gaining increasing attention. It is therefore noteworthy to consider the applicability of organoids for disease modelling and therapeutics. Recently, ground-breaking studies demonstrated that tissue of brain and spinal cord tumours, gliomas, are electrically active and integrated into host neural tissue networks (Venkataramani et al., 2019; Venkatesh et al., 2019). This paved the way for electro-therapies to target gliomas with desirable effects on plasticity and neuromodulation, similar to that observed in brain stimulation for psychiatric and neurological disease (Reviewed in Sprugnoli et al., 2021). Previously, studies found primary-derived glioblastoma cells respond to EF modulation with migration, in a manner highly dependent on the surrounding 2D or 3D microenvironment (Huang et al., 2016). Glioblastoma organoids are producible from genetically-engineered cerebral organoids, and provide an ideal platform for exploration of this phenomena (Bian et al., 2018; Ogawa et al., 2018). However, studies utilising organoids to understand bioelectric-related disease phenotypes are very limited. Using iPSCs derived from schizophrenia patients, schizophrenia-cerebral organoids show an impaired response to electrical stimulation and ion channel-mediated depolarisation when compared to healthy controls (Kathuria et al., 2020). Given the use of deep-brain stimulation to treat schizophrenia and other psychiatric disorders, it would be informative to undertake longitudinal studies examining the response of organoid models of psychiatric disease to electrical stimulation in long-term culture. Moreover, longer-term study of electrical activity may provide deeper insights into the complex mechanism of disrupted bioelectric networks in psychiatric disease, particularly subtle disturbances in EFs which may arise earlier in neurodevelopment, without the need for patient involvement. Nguyen et al. (2018) utilised iPSC-derived human 3D neural progenitor cells to study the effects of stimulation on Rett syndrome. As a rare but debilitating neurodevelopmental disorder, *in vivo* electrical stimulation has not been clinically applied for Rett syndrome patients. However, *via* culturing on conductive graphene scaffolds, the authors demonstrate electrical stimulation of diseased NPCs reduced disease phenotype (Nguyen et al., 2018). In concordance with previous studies, electrical stimulation promoted neurogenesis and neural maturation in Rett disease models, as in wildtype (Nguyen et al., 2018). As molecular responses to EF-modulation are further unveiled, the correlation of these to disease phenotypes may reveal bio-electric related pathologies and uncover new targets for electro-therapy.

## Conclusion and Final Remarks

If we are aiming to truly recapitulate development in the dish, then mapping the bioelectric networks in the body, and in our current organoid cultures *in vitro* is important. Whilst the lack of knowledge of mammalian bioelectricity networks is currently a limiting factor, it does not diminish the utility of EF modulation to enhance organoid development.

In the clinic, the application of EF to the CNS is beneficial (Balossier et al., 2015; Fu et al., 2015; Tao et al., 2016; Marquez-Chin and Popovic, 2020; Krauss et al., 2021; Sui et al., 2021; Karamian et al., 2022). To date, studies of EF modulation *in vitro* hold great promise for increasing the development and functionality of CNS derivative cells. However, they have focused on primary brain-derived cells rather PSC-derived organoids. PSC-derived organoids represent state-of-the-art systems for *in vitro* modelling of CNS organogenesis and disease. EF modulation of PSCs has demonstrated increases in neurogenic differentiation. Next, these findings should be translated into PSC-derived organoid cultures.

Whilst researchers are looking towards implementing advanced technologies to improve organoid differentiations, a simple electrical stimulation culture system could provide an additional, or missing, physiological cue. This could have important implications for increasing the utility of organoids in studies of development and disease, or applications in regenerative medicine and tissue engineering. As a cue for organoid differentiation, electrical stimulation is ready to be implemented in cell biology laboratories. Everything considered, bioelectricity, and the exploration of bioelectric phenomena both *in vivo* and *in vitro* is an exciting area to watch. As a greater understanding of bioelectric phenomena *in vivo* evolves, as will the utility of electrical stimulation as a tool in organoid generation. In parallel, PSC-derived CNS organoids provide the best *in vitro* model to unravel endogenous EFs in the human CNS. Based on the current body of research, we postulate exogenous application of EFs to brain organoids may increase cell proliferation, survival or differentiation of desired cell subtypes, whilst reducing death and necrosis. The optimisation of suitable stimulation parameters and field strength will be key in utilising EF manipulation for engineered brain tissue. Ideally, precise or stage-dependent application of electrical stimulation, or modulation of EFs by other means, could be implemented at key developmental timepoints to control organoid growth and maturation.
